# From Coronavirus Disease 2019 to Herpes Simplex Virus Hepatitis: A Case Report of Complications Linked to Immunosuppressive Therapy

**DOI:** 10.7759/cureus.70931

**Published:** 2024-10-06

**Authors:** Manami Oshiro, Ken Kumagai, Ryo Ito, Maki Kanzawa, Toshinao Itani

**Affiliations:** 1 Department of Gastroenterology, Kobe City Nishi-Kobe Medical Center, Hyogo, JPN; 2 Department of Gastroenterology and Hepatology, Medical Research Institute Kitano Hospital, PIIF Tazuke-Kofukai, Osaka, JPN; 3 Department of Pathology, Kobe City Nishi-Kobe Medical Center, Hyogo, JPN

**Keywords:** acute fulminant hepatitis, covid-19, herpes simplex virus, sars-cov-2, tocilizumab

## Abstract

Severe acute respiratory syndrome coronavirus 2 (SARS-CoV-2) has been linked to severe pneumonia and systemic deterioration in humans. When antiviral drugs and antibodies are not available, it is preferable to choose early treatment methods to suppress cytokine storms. While an interleukin-6 receptor antagonist has proven effective in controlling cytokine storms in coronavirus disease 2019 (COVID-19) pneumonia, it can also increase susceptibility to secondary infections, such as herpes simplex virus (HSV). HSV hepatitis can progress rapidly and be fatal, posing a significant therapeutic challenge. We present the case of a patient with chronic liver disease who developed severe hepatitis following a COVID-19 infection. Despite initial clinical improvement, the patient experienced a relapse marked by high fever, mucosal ulcerations, and deteriorating liver function, eventually leading to acute liver failure and death. Histopathological analysis confirmed HSV hepatitis as the cause of the liver damage. This case underscores the risks associated with HSV reactivation in immunosuppressed patients and highlights the necessity for prompt antiviral treatment and preventive measures. Continuous vigilance is crucial for managing prolonged immunosuppressive states to mitigate severe complications and enhance patient outcomes.

## Introduction

Severe acute respiratory syndrome coronavirus 2 (SARS-CoV-2), which has caused a global pandemic since 2020, leads to severe pneumonia and systemic deterioration in humans. Before the introduction of novel RNA vaccines and antiviral drugs, the primary treatment approach for SARS-CoV-2 infection focused on suppressing cytokine storms. Drawing from previous experience with severe infections, high-dose corticosteroids and immunosuppressants were utilized [1,2]. These treatments are still relevant for managing coronavirus disease 2019 (COVID-19) pneumonia in patients ineligible for vaccination or in high-risk individuals even after vaccination. While immunosuppression is effective in mitigating cytokine storms associated with COVID-19 pneumonia, it also increases susceptibility to secondary infections. Herpes simplex virus (HSV) is particularly problematic in an immunosuppressive state, and HSV hepatitis is a rapidly progressive, life-threatening infection that poses significant treatment challenges. The etiologies of severe acute hepatitis include drug-induced liver injury and infectious agents; however, in cases with rapidly progressing conditions, differentiating HSV hepatitis can sometimes be challenging in practice. Here, we report the case of a patient who developed fatal hepatitis following an initially favorable outcome from a SARS-CoV-2 infection.

## Case presentation

**Figure 1:**
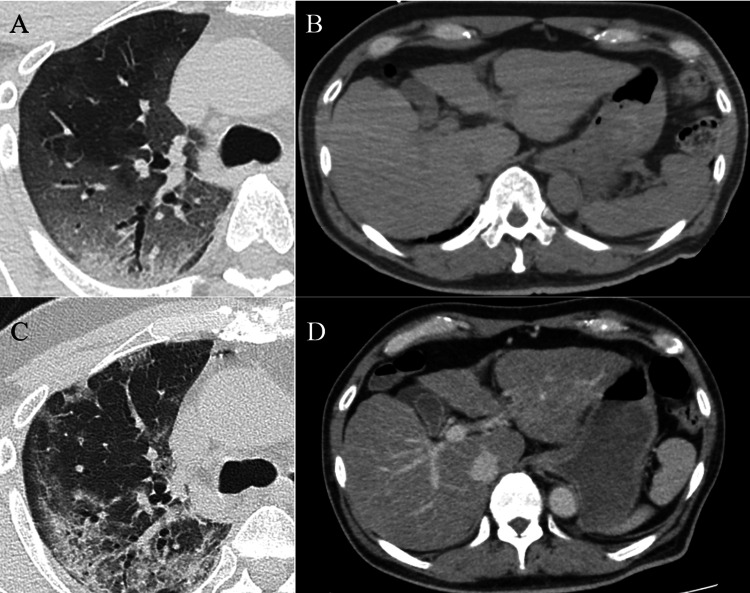
Changes in CT images throughout the clinical course. Chest and abdominal CT images at hospital admission (A, B) and intensive care unit admission (C, D). Ground-glass opacities were diminished and organization was observed (A, C). The gallbladder was edematously thickened, suggesting liver damage such as hepatitis or shock liver. No hepatic atrophy or splenomegaly was noted (B, D).

The patient was a 68-year-old male with a body mass index of 27 kg/m^2^ who had been receiving outpatient care for chronic hepatitis and liver cirrhosis, with a Child-Pugh score of 6. Hepatitis virus infections were not detected, habitual alcohol consumption was absent, and a liver biopsy indicated an unknown etiology, leading to a diagnosis of metabolic dysfunction-associated steatotic liver disease. The patient developed a cough, and four days later, he presented with a fever and tested positive for SARS-CoV-2 via polymerase chain reaction. At that time, RNA vaccines for SARS-CoV-2 had only recently been introduced in Japan, and the patient had not been vaccinated. He received 6 mg of dexamethasone through home care, maintaining an oxygen saturation of 96% with 2 L/minute of supplemental oxygen. His condition worsened with increased chills and dyspnea, leading to his transfer to our hospital due to a drop in SpO_2_ to 93%, despite receiving 3 L/minute of nasal oxygen 11 days after onset. Upon arrival, his vital signs were a pulse rate of 90 beats per minute, blood pressure of 153/85 mmHg, and temperature of 38.3°C. Initial blood tests showed a white blood cell count of 9,900/μL, aspartate aminotransferase (AST) of 43 U/L, alanine aminotransferase (ALT) of 48 U/L, alkaline phosphatase (ALP) of 62 U/L, and lactate dehydrogenase (LDH) of 479 U/L. Due to severe coughing, regular administration of codeine phosphate was initiated. The following day, the patient required 10 L/minute of oxygen via a reservoir, leading to the administration of tocilizumab at a dose of 560 mg and the initiation of as-needed morphine therapy at a dose of 10 mg per dose. According to the COVID-19 pneumonia treatment guidelines in Japan at that time, antiviral drugs were not administered, and the patient continued on oral dexamethasone 6 mg until day 13 of hospitalization. Subsequently, the need for oxygen decreased, and the patient was able to eat while receiving 7 L/minute of oxygen through an oxygen concentrator. Throughout hospitalization, mechanical ventilation was not required, and by day 17 of hospitalization, the patient needed only 1 L/minute of oxygen during rehabilitation. Regular morphine administration continued due to persistent coughing. As the patient’s overall condition and respiratory status improved, preparations were made for transfer to a rehabilitation facility before discharge. On day 19 of hospitalization, the patient developed a fever of 38.3°C, an increased respiratory rate of 30 breaths per minute, and a pulse rate of 130 beats per minute. Oral ulcers, dysphagia, pharyngodynia, and bilateral leg edema worsened, leading to a postponement of discharge. Given the presence of oropharyngeal pain and the suspicion of pharyngitis, treatment with ampicillin/sulbactam was initiated. On day 22 of hospitalization, AST, ALT, ALP, and LDH levels surged to 9,518 U/L, 6,426 U/L, 134 U/L, and 6,814 U/L, respectively, raising suspicion of drug-induced liver injury. Chest CT revealed that the ground-glass opacities had resolved and were replaced by organized shadows, without new pulmonary lesions observed. The gallbladder was edematously thickened, reflecting liver injury, shock liver, or acute heart failure; however, no events suggesting heart failure or sudden hypotension were noted (Figure 1).

Antibiotics were switched to meropenem, and the patient was admitted to the intensive care unit. On day 23 of hospitalization, the patient exhibited signs of drowsiness and had a systolic blood pressure of 60 mmHg. AST, ALT, ALP, and LDH levels further deteriorated to 19,470 U/L, 10,121 U/L, 217 U/L, and 11,485 U/L, respectively. Additionally, the platelet count decreased to 11,000/μL, and the prothrombin time (PT) dropped to 40.6%. Laboratory results are presented in Table 1.

**Table 1:** Laboratory values for blood tests at admission and during hospitalization.

Laboratory results (units)	At admission	Day 12 of hospitalization	Day 20 of hospitalization	Day 22 of hospitalization	Day 23 of hospitalization	Reference range
Hemoglobin (g/dL)	14.8	15.1	14.6	12.8	12.3	13.5–17.0
White blood cell (/μL)	99 × 10^2^	111 × 10^2^	48 × 10^2^	17 × 10^2^	18 × 10^2^	40–90 × 10^2^
Platelets (/μL)	8.9 × 10^4^	13.5 × 10^4^	6.6 × 10^4^	2.1 × 10^4^	1.1 × 10^4^	15.0–35.0 × 10^4^
Creatinine (mg/dL)	0.58	0.58	0.54	0.89	1.41	0.6–1.2
C-reactive protein (mg/dL)	7.1	0.0	0.1	2.6	1.7	0–0.5
Aspartate transaminase (U/L)	43	37	169	9,518	19,470	10–30
Alanine transaminase (U/L)	48	73	273	6,426	10,121	3–30
Alkaline phosphatase (U/L)	62	75	68	134	217	38–113
Lactate dehydrogenase (U/L)	479	427	426	6,814	11,485	124–222
Total protein (g/dL)	7.0	6.1	6.1	5.2	4.8	6.7–8.3
Albumin (g/dL)	3.5	3.4	3.6	2.8	2.5	3.8–5.1
Total bilirubin (mg/dL)	0.8	1.4	0.9	1.4	1.8	0.2–1.2
Direct bilirubin (mg/dL)	-	-	-	0.6	1.1	0.1–0.5
Blood urea nitrogen (mg/dL)	20	39	19	29	44	8–20
Prothrombin time (%)	-	-	-	-	40.6	80–125
International normalized ratio	1.1	1.1	1.1	1.3	1.5	0.9–1.4

**Figure 2:**
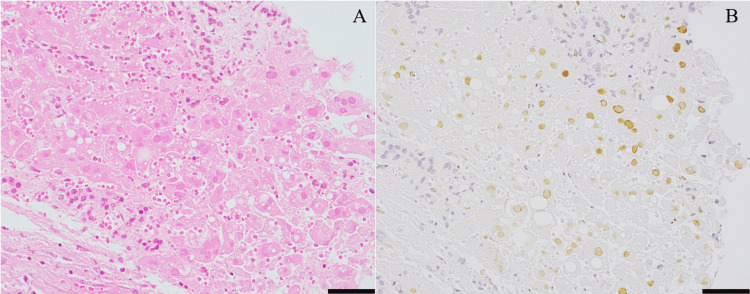
Histopathological images of the liver biopsy. Histopathological images stained with hematoxylin and eosin (A) and with anti-herpes simplex virus 1 antibody (B). The scale bar indicates 50 μm.

The patient was diagnosed with acute-on-chronic liver failure and was promptly initiated on treatment with 1,000 mg of methylprednisolone and plasma exchange. Although liver transplantation is a commonly utilized intervention for acute liver failure in Japan, the presence of immunosuppression secondary to tocilizumab, coupled with the deteriorated respiratory status resulting from COVID-19 pneumonia, led the attending physicians at the specialized facility to conclude that the patient was not a suitable candidate for liver transplantation in this instance. This decision was predicated on the elevated risk of infection associated with additional immunosuppression post-transplant. On the same day, coagulation abnormalities and acidemia progressed, with a Mayo End-Stage Liver Disease score of 27 and meeting the criteria for disseminated intravascular coagulation (DIC), leading to the administration of recombinant thrombomodulin and platelet transfusion. On day 24 of hospitalization, antithrombin III supplementation and rifaximin were initiated, but the patient succumbed to his condition on day 26 of hospitalization. Due to restrictions on autopsies during the SARS-CoV-2 pandemic, a liver biopsy was performed as a necropsy. Histopathological examination revealed extensive coagulative necrosis with very few viable hepatocytes. Hepatocytes with preserved nuclei exhibited glassy changes and chromatin displacement to the nuclear periphery. No Owl-eye intranuclear inclusions indicative of cytomegalovirus (CMV) infection were observed, and granulomas were absent. Proliferation of atypical cells was not noted, and inflammation was minimal. The portal areas showed only mild fibrosis with the presence of bile ducts and blood vessels. Based on hematoxylin and eosin staining, HSV infection was suspected. Immunohistochemistry confirmed HSV type 1 positivity in the nuclei of necrotic and severely degenerated hepatocytes (Figure 2), while CMV and Epstein-Barr virus (EBV) staining were negative.

Thus, the liver tissue was diagnosed with HSV hepatitis. Reactivation of HSV in the context of immunosuppression likely led to severe and irreversible hepatocellular necrosis.

## Discussion

During the period when antiviral drugs and vaccines were unavailable, immunosuppressive agents were employed to manage inflammation in COVID-19 pneumonia. Currently, dexamethasone and tocilizumab, an interleukin-6 (IL-6) receptor antagonist, remains a viable therapeutic option for preventing critical illness with hypoxic respiratory failure due to excessive immune responses [1,2]. IL-6 is a key mediator in cytokine storms, and tocilizumab continues to play a crucial role in the management of COVID-19 pneumonia by broadly suppressing the immune response. In this case, tocilizumab administration improved oxygenation and is believed to have mitigated the cytokine storm, enhancing pulmonary ventilation and contributing to the patient’s recovery to a dischargeable state. Tocilizumab is also used in autoimmune conditions characterized by excessive immune responses; however, its potent effects come with an increased susceptibility to infections. In autoimmune diseases such as rheumatoid arthritis, reactivation of viruses such as CMV, EBV and varicella-zoster virus (VZV) can occur [3,4]. HSV is another virus of concern, with reactivation being a notable issue not limited to tocilizumab but applicable to immunosuppressive states in general. HSV remains latent in immunocompetent individuals but can cause mucosal lesions or, in severe cases, liver damage and encephalitis when host immunity is compromised [5]. IL-6 plays a role in infection control through various mechanisms [6], and its inhibition can impair HSV control, potentially leading to viral reactivation. In this case, HSV reactivation resulted in acute liver failure; however, the liver was not the sole organ affected. The rapid decline in platelet count observed cannot be fully explained by the limited lifespan of platelets alone. It is postulated that sepsis, whether viral or bacterial, activates platelets and leads to DIC [7], indicating that the patient was experiencing viremia. Although the presence of pancytopenia and fever raises the possibility of hemophagocytic lymphohistiocytosis (HLH), the diagnosis was not confirmed according to the HLH-2004 criteria [8]. There is established awareness that conditions requiring chronic immunosuppression, such as autoimmune diseases and post-transplant states, are associated with an increased risk of infection. However, in scenarios such as COVID-19 pneumonia, where tocilizumab is used transiently, there may be a lack of awareness regarding the implications of sustained immunosuppression [9]. Given that the combination of tocilizumab and steroids induces a significant immunosuppressive state, long-term monitoring for potential organ damage due to HSV is essential, and its use should be limited to severe COVID-19 cases. However, even if antiviral treatment is initiated after the diagnosis of HSV infection, there may be cases where survival is not achievable [10]. Initiating treatment without waiting for test results could potentially enhance the survival rate. Nonetheless, there is no consensus on the optimal duration of prevention, underscoring the need for further research.

## Conclusions

This case report highlights the significant risks associated with the combination therapy of tocilizumab and corticosteroids in the management of COVID-19, particularly the potential for severe secondary infections such as HSV hepatitis. Despite an initial favorable clinical response to tocilizumab and corticosteroids, the patient developed fatal HSV hepatitis, illustrating the grave consequences of viral reactivation in an immunosuppressive state. This underscores the importance of vigilant monitoring for latent infections and considering antiviral prophylaxis in patients receiving immunocompromised treatment. Furthermore, it emphasizes the necessity of preventive strategies to mitigate the risks associated with immunosuppression. It is paramount to balance the therapeutic benefits of such regimens with their inherent risks. Future research should prioritize the optimization of management protocols to improve patient safety and outcomes in the context of prolonged immunosuppressive states.
